# Predictors of Morphine Efficacy for Dyspnea in Inpatients with Chronic Obstructive Pulmonary Disease: A Secondary Analysis of JORTC-PAL 07

**DOI:** 10.1089/pmr.2020.0078

**Published:** 2021-01-07

**Authors:** Yoshinobu Matsuda, Tatsuya Morita, Hirotaka Matsumoto, Keita Hosoi, Kayo Kusama, Yasuo Kohashi, Hiroshi Morishita, Sawako Kaku, Keisukie Ariyoshi, Shunsuke Oyamada, Yoshikazu Inoue, Satoru Iwase, Takuhiro Yamaguchi, Mitsunori Nishikawa

**Affiliations:** ^1^ Department of Psychosomatic Internal Medicine, National Hospital Organization Kinki-Chuo Chest Medical Center, Sakai, Japan.; ^2^ Clinical Research Center National Hospital Organization Kinki-Chuo Chest Medical Center, Sakai, Japan.; ^3^ Palliative and Supportive Care Division, Seirei Mikatahara General Hospital, Hamamatsu, Japan.; ^4^ Department of Respiratory Medicine, Hyogo Prefectural Amagasaki General Medical Center, Aamagasaki, Japan.; ^5^ Department of Respiratory Medicine, Itami City Hospital, Itami, Japan.; ^6^ Department of Respiratory Medicine, Sakai City Medical Center, Sakai, Japan.; ^7^ Department of Respiratory Medicine, HARUHI Respiratory Medical Hospital, Kiyosu, Japan.; ^8^ Department of Respiratory Medicine, Osaka Habikino Medical Center, Habikino, Japan.; ^9^ Department of Diagnostic Radiology, National Cancer Center Hospital, Tokyo, Japan.; ^10^ Department of Data Management, JORTC Data Center, Tokyo, Japan.; ^11^ Department of Biostatistics, JORTC Data Center, Tokyo, Japan.; ^12^ Department of Palliative Medicine, Saitama Medical University, Moroyama, Japan.; ^13^ Division of Biostatistics, Tohoku University Graduate School of Medicine, Sendai, Japan.; ^14^ Department of Palliative Care, National Center for Geriatrics and Gerontology, Obu, Japan.

**Keywords:** COPD, dyspnea, morphine, opioid treatment, respiratory illness

## Abstract

***Objective:*** This study aimed to explore the predictors of morphine efficacy in the alleviation of dyspnea in COPD.

***Background:*** Dyspnea is prevalent in patients with chronic obstructive pulmonary disease (COPD) and often persists despite conventional treatment.

***Methods:*** A secondary analysis of a multi-institutional prospective before–after study was conducted focusing on morphine use for alleviating dyspnea in COPD patients. Subjects included COPD patients with dyspnea at seven hospitals in Japan. Patients received 12 mg/day of oral morphine (or 8 mg/day if they had low body weight or renal impairment). Univariate and multivariate logistic regression analyses were performed with numerical rating scale (NRS) score of the current dyspnea intensity in the evening of day 0, Eastern Cooperative Oncology Group Performance Status (ECOG PS; ≤2 or ≥3), age, and partial arterial pressure of carbon dioxide (PaCO_2_) as independent factors; an improvement of ≥1 in the evening NRS score of dyspnea from day 0 to 2 was the dependent factor.

***Results:*** Thirty-five patients were enrolled in this study between October 2014 and January 2018. Excluding one patient who did not receive the treatment, data from 34 patients were analyzed. In the multivariate analysis, lower PaCO_2_ was significantly associated with morphine efficacy for alleviating dyspnea (odds ratio [OR] 0.862, 95% confidence interval [CI] 0.747–0.994), whereas the NRS of dyspnea intensity on day 0 (OR 1.426, 95% CI 0.836–2.433), ECOG PS (OR 4.561, 95% CI 0.477–43.565), and patients' age (OR 0.986, 95% CI 0.874–1.114) were not.

***Discussion:*** Morphine can potentially alleviate dyspnea in COPD patients with lower PaCO_2_. Trial registration: UMIN000015288 (http://www.umin.ac.jp/ctr/index.htm)

## Introduction

Chronic obstructive pulmonary disease (COPD) is an common lung disease predominantly caused by smoking tobacco.^1^ COPD is the fourth cause of death in the world.^2^ In Japan, 5.3 million people >40 years and 2.1 million people >70 years have COPD.^3^ A majority of COPD patients suffer from dyspnea, which is the most prevalent symptom during the past few months before death.^4,5^ In addition to being a distressing symptom for COPD patients,^6^ dyspnea is linked to increased anxiety and depression, and has a negative impact on health-related quality of life.^7–10^ Despite the availability of treatments such as bronchodilators, inhaled corticosteroids, oxygen therapy, and pulmonary rehabilitation,^11^ dyspnea persists in patients with COPD.

Opioids have been reported to be effective to relieve dyspnea in patients with COPD.^12^ In fact, COPD guidelines suggest opioids improve dyspnea.^13–16^ A multi-institutional prospective before–after original study on the effect of oral morphine on Japanese COPD patients who suffered from dyspnea at rest revealed that morphine had a positive effect on dyspnea. However, some previous studies have reported possible adverse events arising from the use of opioids to treat patients with COPD.^17,18^ In addition, a randomized control trial conducted on patients with chronic breathlessness syndrome failed to prove the efficacy of morphine for the treatment of dyspnea.^19^ Identifying predictors of the response to morphine can help us personalize symptom management and provide better patient-centered care. In this study, we explored potential factors that could predict the responses to morphine use for alleviating dyspnea in patients with COPD.

## Methods

Our protocol was reviewed and approved by the JORTC Protocol Review Committee and the Institutional Review Boards associated with each of the study sites.

### Study design and setting

The JORTC-PAL07 study was conducted between October 2014 and February 2018 with 35 COPD patients from seven hospitals in Japan. Eligibility criteria were described in a previously published report.^20^ Key inclusion criteria were as follows: inpatients who (1) were diagnosed with COPD using the GOLD 2014 diagnostic criteria; (2) had resting dyspnea despite conventional COPD treatment; (3) were ≥40 years; (4) had a smoking history of 10 pack-years or more; (5) had blood oxygen saturation levels (SpO_2_) ≥90% with or without oxygen within 14 days before enrollment and partial pressure of carbon dioxide (PaCO_2_) ≤60 Torr within 28 days before enrollment; and (6) were expected to survive for at least a month. Key exclusion criteria were as follows: patients who (1) had contraindications for morphine; (2) had used any opioid; (3) had pulmonary pathology other than COPD causing dyspnea.

### Procedure

The procedure of the original study has been detailed in a previously published report.^20^ In brief, a multi-institutional prospective before–after study on oral morphine use in Japanese COPD patients with dyspnea at rest was conducted. A dose of 12 mg of morphine was administered to patients (or a reduced dose of 8 mg if their body weight was <40 kg or their estimated glomerular filtration rate was <60 mL/min).^21^ As sustained-release morphine is deemed an off-label drug for noncancer patients in Japan, the patients received 3 mg (or 2 mg) of immediate-release morphine every six hours. Patients' medical records were reviewed to obtain baseline data within two weeks before enrollment.

Current intensity of dyspnea was assessed using an 11-point Likert-type numerical rating scale (NRS) from day 0 to 2 in the mornings (09:00 ± 1 h) and evenings (15:00 ± 1 h).^22,23^ Vital signs and opioid-related adverse events were recorded on days 0–2 in the evening. The Richmond Agitation and Sedation Scale was administered on days 0 and 2 in the evening. Adverse events, based on the Common Terminology Criteria for Adverse Events V.4.0, were assessed in the evenings on days 1 and 3. The study was performed in accordance with the Declaration of Helsinki and the Japanese ethical guidelines for clinical research.

### Statistical analysis

We analyzed the descriptive statistics for baseline patient characteristics. The primary outcome of the original study was a change in the evening NRS score of dyspnea from day 0 to 2. As a change of 1 on the NRS represents a clinically important difference in chronic breathlessness, it was determined that an improvement of 1 point or more would indicate that morphine is an effective treatment for dyspnea in COPD patients.^24^

Therefore, patients' response to the administration of morphine for dyspnea in this secondary analysis was also defined as showing a reduction of ≥1 in the evening NRS from day 0 to 2. Univariate and multivariate logistic regression analyses were performed using the forced entry methods with the NRS score of dyspnea intensity on the evening of day 0, the Eastern Cooperative Oncology Group Performance Status (ECOG PS) (≤2 or ≥3), patient age, and PaCO_2_ as the independent factors, and the response of the patient to the morphine administered for dyspnea as the dependent factor. The four independent factors were selected based on a literature review as follows as well as a discussion among the investigators. The NRS score of dyspnea and patient age were selected based on a previous study.^25^ ECOG PS was selected as an independent factor because previous reports had identified a high ECOG PS score as a possible predictor of the efficacy of opioids in the treatment of refractory breathlessness.^26^ Finally, we selected PaCO_2_ as an independent factor because hypercapnia is one of the possible causes of dyspnea and we hypothesized that patients with hypocapnia would respond to morphine to treat dyspnea.^27^ We determined PaCO_2_ as continuous variable because of the exploratory nature of our study. We also conducted a sensitivity analysis using PaCO_2_ as a binary variable (i.e., ≤45 or >45 Torr). This cutoff point was decided based on a similar cutoff point of PaCO_2_ used for type 2 chronic respiratory failure in Japan.^28^ A two-sided significance level of 0.05 was used for the univariate and multivariate logistic regression analyses. All statistical analyses were performed using SAS version 9.4 (SAS Institute, Cary, NC, USA).

## Results

### Patients

The mean age of the patients was 72.9 ± 8.9 years; 94.3% were male; 83% had modified Medical Research Council scale ratings of 3 or 4; and 71.4% received oxygen therapy (Table 1).

**Table 1.** Baseline Patient Characteristics

	n = 35
Age (years), mean (SD)	72.9 (8.9)
Male gender, *n* (%)	33 (94.3)
ECOG performance status, *n* (%)	
0–2	20 (57.1)
3–4	15 (42.9)
History of smoking, *n* (%)	
Former smoker	32 (91.4)
Current smoker	2 (5.7)
Smoking (pack-years), median (IQR)	55.5 (42.0–80.0)
Modified MRC dyspnea scale, *n* (%)	
0–2	6 (17.1)
3–4	29 (82.9)
NRS score of dyspnea intensity in the evening of day 0, *n* (%)	
1–3	18 (51.4)
4–6	10 (28.6)
7–10	7 (20.0)
NRS score of dyspnea intensity in the evening of day 0, median (IQR)	3.0 (2.0–6.0)
GOLD stage, *n* = 29	
I	2 (6.9)
II	3 (10.3)
III	12 (41.4)
IV	12 (41.4)
Total CAT score, mean (SD)	23.1 (6.1)
BMI (kg/m^2^), mean (SD)	20.4 (3.8)
Postbronchodilator pulmonary function^a^	
FEV_1_ (L), mean (SD), *n* = 31	0.87 (0.52)
% FEV_1_ (%), mean (SD), *n* = 29	37.3 (20.4)
FEV_1_/FVC (%), mean (SD), *n* = 31	38.9 (13.1)
PaCO_2_ (Torr), mean (SD)	44.0 (8.1)
≥45	14 (40.0)
<45	21 (60.0)
eGFR (mL/min), mean (SD)	77.7 (21.5)
Oxygen delivery device, *n* (%)	
Nasal cannula	23 (65.7)
Oxymizer^®^	2 (5.7)
Mask	0 (0)
Medication for COPD, *n* (%)	
Long-acting muscarinic antagonist	27 (77.1)
Long-acting β_2_-agonist	28 (80.0)
Inhaled corticosteroid	21 (60.0)

^a^ Data within one year before enrollment.

BMI, body mass index; CAT, COPD assessment test; COPD, chronic obstructive pulmonary disease; ECOG, Eastern Cooperative Oncology Group; eGFR, estimated glomerular filtration rate; FEV_1_, forced expiratory volume in one second; FVC, forced vital capacity; GOLD, global initiative for chronic obstructive lung disease; MRC, medical research council; NRS, numerical rating scale; PaCO_2_, arterial partial pressure of carbon dioxide.

### Changes NRS score of dyspnea intensity

The NRS score of dyspnea intensity in the evening significantly decreased from 3.0 on day 0 (95% confidence interval [CI] 3.1–4.8) to 2.4 on day 2 (95% CI 1.7–3.1; *p* = 0.0002). Twenty-six patients (76.5%) recorded a decrease of 1 point or more in the NRS score from day 0 to 2, and 8 patients (23.5%) were classified as nonresponders.

### Adverse events

There were no apparent changes in the mean scores of opioid-related adverse events and vital signs. One patient experienced grade 3 lung infection not associated with morphine.

### Factors predicting the response to morphine for dyspnea

In the univariate logistic regression analysis, lower PaCO_2_ was significantly associated with response to morphine (odds ratio [OR] 0.889, 95% CI 0.792–0.997), whereas the NRS score of dyspnea intensity on day 0 (OR 1.446, 95% CI 0.908–2.301), ECOG PS (OR 2.571, 95% CI 0.435–15.193), and patient age (OR 0.983, 95% CI 0.897–1.077) were not significantly associated with a response to morphine (Table 2). A sensitivity analysis using PaCO_2_ as a binary variable revealed that PaCO_2_ ≤45 was significantly associated with patients' responses to morphine administered to alleviate dyspnea (OR 0.123, 95% CI 0.020–0.758).

**Table 2.** Univariate Logistic Regression Analysis

Predictors	OR	95% CI	p
NRS of dyspnea intensity on day 0	1.446	0.908–2.301	0.120
ECOG PS (≤2 or ≥3)	2.571	0.435–15.193	0.297
Age	0.983	0.897–1.077	0.711
PaCO_2_	0.889	0.792–0.997	0.045

ECOG PS, Eastern Cooperative Oncology Group Performance Status; NRS, numerical rating scale; OR, odds ratio.

In the multivariate logistic regression analysis, lower PaCO_2_ was significantly associated with the efficacy of morphine use for alleviating dyspnea (OR 0.862, 95% CI 0.747–0.954), but NRS score of dyspnea intensity on day 0 (OR 1.426, 95% CI 0.836–2.433), ECOG PS (OR 4.561, 95% CI 0.477–43.565), and patient age (OR 0.986, 95% CI 0.874–1.114) did not show this association (Table 3). In the sensitivity analysis using PaCO_2_ as a binary variable, PaCO_2_ ≤45 was significantly associated with patients' responses to morphine use for alleviating dyspnea (OR 0.074, 95% CI 0.008–0.705; Supplementary Table S1).

**Table 3.** Multivariate Logistic Regression Analysis

Predictors	OR	95% CI	p
NRS of dyspnea intensity on day 0	1.426	0.836–2.433	0.193
ECOG PS (≤2 or ≥3)	4.561	0.477–43.565	0.188
Age	0.986	0.874–1.114	0.824
PaCO_2_	0.862	0.747–0.994	0.041

## Discussion

In this secondary analysis of data from a multi-institutional prospective before–after study, lower PaCO_2_ was associated with a response to morphine for the alleviation of dyspnea (OR 0.862, 95% CI 0.747–0.954), based on multivariate logistic regression analysis. A sensitivity analysis using a binary PaCO_2_ variable yielded the same results.

Lower PaCO_2_ could reflect a better respiratory function and an earlier phase of the disease trajectory compared with a high PaCO_2._^29^ Dyspnea in COPD patients generally develops in the terminal stage when morphine is used to alleviate it.^5^ However, the results from this study indicate that morphine could also be useful in alleviating dyspnea in the early stages.

Johnson et al. conducted a multicenter retrospective analysis of 213 individual pooled datasets from four clinical trials on the use of an opioid for chronic refractory breathlessness.^25^ They reported that higher breathlessness and younger age predicted the efficacy of the opioid in the treatment of breathlessness. However, they did not assess the impact of PaCO_2_ in their study. To our knowledge, our study was the first to investigate PaCO_2_ as a factor predicting the response to morphine used to alleviate dyspnea in patients with COPD. To confirm the association, PaCO_2_ should be assessed at baseline to confirm the relationship between PaCO_2_ and the response to morphine for dyspnea in future studies. The NRS score of dyspnea intensity on day 0 and patient age were not associated with the efficacy of morphine as a treatment for dyspnea in our study. A small sample size and a narrow distribution of these variables could be potential explanations for these results.

Based on our findings, conducting arterial blood gas analysis to assess PaCO_2_ might be appropriate to predict the effectiveness of morphine for dyspnea in patients with COPD. However, arterial puncture may cause some complications, such as pain, bleeding, and neuropathy.^30^ Therefore, it is important to consider the balance of risks and benefits for each patients when deciding to conduct arterial puncture before using morphine, especially in palliative care settings. In addition, conducting arterial blood analysis may be limited to inpatient setting. In outpatient setting, just trialing whether morphine is effective may be reasonable. Considering these things, it may be reasonable to check the recent PaCO_2_ data already measured if available.

This study had some limitations. First, this was a before–after study; therefore, the efficacy of morphine was investigated not in terms of change in the level of dyspnea between the group treated with morphine and a placebo group in a randomized control study, but in terms of a change in the level of dyspnea from the baseline to day 2. In addition, a placebo could have overestimated the efficacy of morphine in the treatment of dyspnea among COPD patients. Second, the small sample size could have caused us to overlook some important morphine efficacy predictors. Third, we conducted this study in an inpatient setting. Thus, we need to investigate the predictors of the response to morphine in outpatients setting in the future, where arterial blood gas analysis is unlikely to be available. Fourth, we excluded patients with PaCO_2_ >60 Torr; however, these patients have most severe dyspnea and require the management for it. Fifth, the dose of morphine (12 or 8 mg a day) was lower than in previous studies^19,31^ although dyspnea was significantly improved by this dose of morphine in our study. Sixth, we assessed the efficacy of the treatment only 2 days after its initiation. This is because we believed a longer study period would have been less feasible, as COPD patients were vulnerable, and their fragile condition could warrant a change in their treatment, including the use of supplemental oxygen or administration of an inhaled bronchodilator. Factors associated with long-term efficacy of the use of morphine in the treatment of dyspnea should be addressed in future studies. Finally, we did not record comorbid conditions. Although we excluded patients with pulmonary pathology other than COPD causing dyspnea, other conditions that could contribute to dyspnea such as heart failure were not analyzed.

## Conclusions

Inpatients with lower PaCO_2_ may respond particularly well to initiation of morphine use for relief of dyspnea; in contrast, the baseline NRS score of dyspnea intensity, performance status, and patient age were not significantly associated with improvement of dyspnea. A large study is needed in the future to confirm the relationship between response to morphine for dyspnea and low PaCO_2_ in COPD patients.

## Supplementary Material

Supplemental data

## Abbreviations Used

BMI: body mass index
CI: confidence interval
COPD: chronic obstructive pulmonary disease
ECOG PS: Eastern Cooperative Oncology Group Performance Status
eGFR: estimated glomerular filtration rate
NRS: numerical rating scale
OR: odds ratio
PaCO_2_: partial arterial pressure of carbon dioxide
SD: standard deviation
