# Supplementary material for: Predictors of Morphine Efficacy for Dyspnea in Inpatients with Chronic Obstructive Pulmonary Disease: A Secondary Analysis of JORTC-PAL 07
Source: Palliat Med Rep. 2021 Jan 7;2(1):15–20. doi: 10.1089/pmr.2020.0078 (PMC8241369; doi:10.1089/pmr.2020.0078)
Supplement: Supplemental data [file Supp_Table1.docx]

| **SUPPLEMENTARY TABLE 1. MULTIVARIATE LOGISTIC REGRESSION ANALYSIS** | | | |
| --- | --- | --- | --- |
| Predictors | Odds ratio | 95% CI | P-value |
| NRS of dyspnea intensity on Day 0 | 1.310 | 0.812 – 2.114 | 0.268 |
| ECOG PS (≤ 2 or ≥ 3) | 5.353 | 0.534 – 53.655 | 0.154 |
| Age | 0.985 | 0.868 – 1.119 | 0.822 |
| PaCO_2_ (≤ 45 Torr or > 45 Torr) | 0.074 | 0.008 – 0.705 | 0.024 |

NRS numerical rating scale; ECOG PS Eastern Cooperative Oncology Group Performance Status.
